# To determine the effect of wearing shoe covers by medical staff and visitors on infection rates, mortality and length of stay in Intensive Care Unit

**Published:** 2014

**Authors:** Zeeshan Ali, Aayesha Qadeer, Aftab Akhtar

**Affiliations:** 1 Dr. Zeeshan Ali, MBBS, FCPS Med, Fellow Critical Care, Shifa International Hospital, Islamabad, Pakistan.; 2 Dr. Aayesha Qadeer, MBBS, Senior Medical Officer Medical ICU, Shifa International Hospital, Islamabad, Pakistan.; 3 Dr. Aftab Akhtar, MBBS, MD. USA, Head of Department and Consultant Pulmonology and Critical Care, Shifa International Hospital, Islamabad, Pakistan.

**Keywords:** ICU, Intensive Care Unit, Shoe Covers, mortality, Length of ICU stay

## Abstract

***Objective:*** Intensive Care Units (ICUs) experience higher infection rates due to the severity of illness and frequent use of invasive devices. Use of personal protective equipment reduces the risk of acquiring an infection. This study has been conducted to determine the role of using shoe covers by medical staff and visitors on infection rates, mortality and length of stay in ICU.

***Methods:*** It is a descriptive study, performed in Shifa International Hospital, Islamabad from January 2012 to July 2012. The rates of infection (by checking patients for common ICU pathogens), mortality and length of stay of patients admitted in MICU and SICU from January 2012 to March 2012 were measured. Use of shoe covers was abandoned during this period. The same parameters were measured for the patients admitted from May, 2012 to July, 2012; the period during which shoe covers were strictly used by all the staff members and visitors. The data was then analyzed and compared using chi-square test with significance value at p< 0.05.

***Results:***A total of 1151 patients were studied in 06 months period. Among the two groups of patients, managed with and without using shoe covers in ICU, statistically significant decrease was seen in terms of length of ICU stay(as P value is less than 0.05) in patients managed in duration of shoe covers. However, the time period in which shoe covers were used the infections with three common ICU pathogens MRSA, VRE and acinetobacter were statistically significant more than the periods in which shoe covers were not used. There was no significant difference in mortality for both groups (P value = 0.146).

***Conclusion:*** Use of shoe covers in critical care area is not helpful in preventing infections of common ICU pathogens and length of stay in ICU patients; nor has it decreased the mortality.

## INTRODUCTION

Large hospital-wide infection prevention schemes, focusing largely on increased awareness and improved hand-hygiene, has emphasized upon limiting the development of nosocomial infections.^1^ Three organisms are common in hospital settings; the ICU patients are especially vulnerable to these infections. Acinetobacter baumannii is a Gram negative bacteria. It can be an opportunistic pathogen in humans, affecting people with compromised immune system. It has been isolated from soil and water samples in the environment.^2^ MRSA and VRE can cause invasive and life-threatening infections, such as osteomyelitis, bacteremia, endocarditis, pneumonia, urinary tract infections, intra-abdominal or pelvic infections, vascular line sepsis, and wound and surgical infections. The primary route of transmission of MRSA from patient to patient is via contaminated hands of healthcare workers but it is not known that whether shoe dusts spread the infection or not.^3^ However, Gram negative infections are mainly spread from the endogenous source but cross transmission is being recognized. Current guidelines for the prevention of spread of multi-resistant bacteria in the hospital setting do not distinguish between Gram-positive and Gram-negative isolates.^4^

Colonization refers to the presence of microorganisms in or on a host with growth and multiplication, but without tissue invasion or damage. Infection is the entry and multiplication of microorganisms in the tissues of the host leading to local or systemic signs and symptoms of infection.^5^

Using personal protective equipment provides a physical barrier between micro-organisms and the wearer. It offers protection by helping to prevent microorganisms from contaminating hands, eyes, clothing, hair and shoes; hence preventing transmission to other patients and staff.^2^Personal protective equipment includes: Gloves, protective eye wear, Masks, Apron Gown, Shoe covers and a Cap / Haircover.^6^

Personal protective equipment reduces but does not completely eliminate the risk of acquiring an infection. It is important to use it effectively, correctly, and at all times when contact with blood and body fluids of the patients may occur. Staff must also be aware that use of personal protective equipment does not replace the need to follow basic infection control measures such as hand hygiene.^7^

Personal protective equipment should be chosen in accordance with the risk of exposure. Health care workers should assess whether they are at risk of exposure to blood, body fluids, excretions or secretions and choose their items of personal protective equipment according to the assessed risk.^8^ Disposable caps and protective foot wear should be used where there is a likelihood that patient’s blood, body fluids, secretions or excretions might splash, spill or leak onto the hair or shoes.^6^^-^^8^

Shoe covers should not however be used for the prevention of surgical site infections (nevertheless, shoe covers are required by OSHA regulations when "gross contamination can reasonably be anticipated") (CDC category IB) A-IV.^9^ The use of shoe covers has never been proven to decrease the risk or incidence of surgical site infections, or to decrease the bacterial counts of the operating room floors.^10^^,^^11^ It also has no mortality benefit when compared to unrestricted access to surgical ICU.^12^

Our study measures the advantage of using shoe covers in intensive care setup in terms of rate of infection, mortality and length of ICU stay.

## METHODS

It was a prospectively conducted study in Medical and Surgical ICU of Shifa International Hospital, Islamabad. Total duration of study was 06 months. Two groups were made. The first group included patients who were managed in the above mentioned ICU’s when all the staff members and the visitors were not using shoe covers while being in ICU i.e. from January to March 2012. The other group included the patients, who were given critical care when shoe covers were strictly being used by the staff and the visitors in the ICU i.e. from May to July, 2012. The parameters studied in all the patients were the rate of infections, mortality and length of ICU stay to assess whether wearing shoe covers would reduce infections and improve these parameters.

All the patients presenting in Medical and Surgical ICU in the above mentioned 06 months were included in the study. From January 2012 to March 2012, when shoe covers were not in use, other infection control practices such as hand washing and antisepsis, use of personal protective equipment when handling blood, body substances, excretions and secretions, environmental cleaning and spills-management and appropriate handling of waste, remained the same as for the other group. During May, 2012 to July, 2012; the period during which shoe covers were being used, none of the staff members, consultants, residents, fellows, nurses, paramedical staff and visitors were allowed in the ICU’s without shoe covers. A boundary line was drawn and a warning note was pasted at the ICU entrance regarding strict use of shoe covers. Infection control nurses and ICU staff scrutinized every person.

The patients were then serially followed for their length of stay, mortality and incidence of infections with three common ICU pathogens Acinetobacter, MRSA and Vancomycin resistant Enterococcus by obtaining cultures from blood, urine, sputum and other body secretions.

Data was entered and analyzed using SPSS version 16.0. Descriptive statistics were calculated. Chi-square test was applied to determine the difference in infection, mortality and length of stay in two groups. P value of less than 0.05 is considered significant.

## RESULTS

A total of 1151 patients were admitted in medical and surgical ICU of shifa International hospital, Islamabad during the 06 months period of this study. All were included in the study. Out of these 63.7% (733) were below 60 years of age while the remaining 36.3% (418) were 60 years or more of age. 64% (737) patients were males and 36% (414) patients were females. Among the total 1151, 51.9% (597) were admitted in MICU whereas 48.1% (554) were admitted in SICU. 55.4%(638) patients were admitted in the duration when shoe covers were not being used while 44.6% (513) patients were admitted in the three months when shoe covers were being used by MICU and SICU staff and visitors. The cultures were done for common ICU pathogens (acinetobacter, vancomycin resistant Enterococcus, MRSA) and only 6.6% (76) patients were found to be culture positive for these organisms. Acinetobacter was obtained in tracheal aspirates of 36 patients, while MRSA was found in 12 patients as shown in Bar chart 1. There were a total of 20.7% (238) deaths during the study period. The duration of ICU stay was within three days for 61.8% (711) patients whereas 17.3% (199) patients stayed for more than six days (Table-I).

Comparing the culture positive patients, out of total 6.6% (76) culture positive patients 2.8% (32) were from MICU and 3.8% (44) were from SICU, while 30 patients were culture positive before the use of shoe covers and 46 patients came out to be culture positive when shoe covers were in use, p value is 0.004 as shown in Table-I. Among the total 20.7% (238) deaths, 10.6% (122) occurred in the period when shoe covers were not being used while 10.1% (116) were seen in the time period when shoe covers were being used (p value >0.05) (Table-I). More number of MICU patients expired i.e. 14.1% (162) when compared to SICU patients, where only 6.6% (76) deaths were observed. The p value came out to be 0.001. Considering the length of stay, 65% (100) patients stayed in ICU before using the shoe covers while 57.7% (99) patients stayed in ICU for less than 03 days during the time when shoe covers used (p value <0.05) as seen in Table-I.

**Table-I:** Rates of Infection, Mortality and length of stay before and after use of shoe cover

	*Before shoe cover*	*After Shoe cover*	* P value*
*Infections*			
Yes	30 (2.6%)	46 (4.0%)	0.004
No	608 (52.8%)	467 (40.6%)	
*Mortality*			
Yes	122 (10.6%)	116 (10.1%)	0.146
No	516 (44.8%)	397 (34.5 %)	
*Length Of stay*			
1-3 days	415 (65%)	296 (57.7%)	0.038
days	123 (19.3%)	118 (23%)	
>6days	100 (15.7%)	99 (19.3%)	

**Bar Chart 1:**
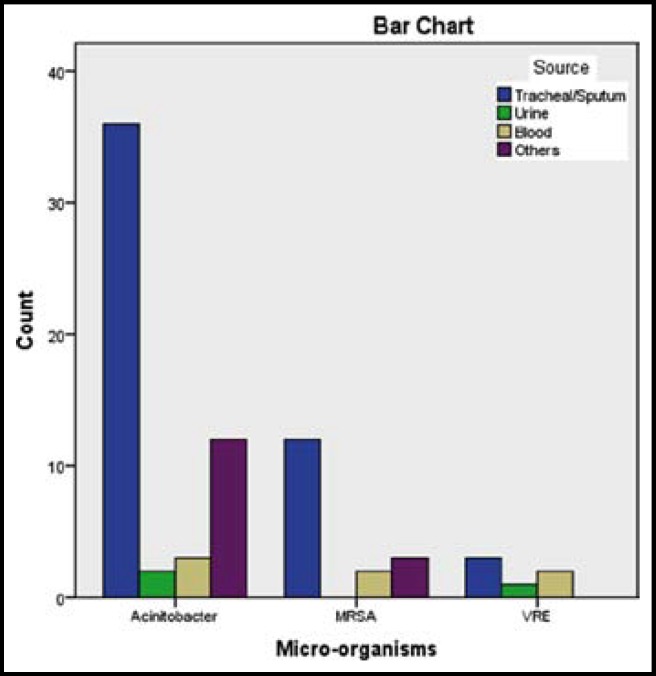
Number of patients infected and Microorganisms obtained from various sources in these patients

## DISCUSSION

Intensive care unit (ICU) is one of the most eventful units of the hospital and uses sophisticated equipment and advanced medical practices. At the same time ICU may experience higher infection rates due to the severity of illness and frequent use of invasive devices such as intravenous catheters, feeding tubes, airways, etc. Not only are there chances for infection from patients’ own endogenous microorganisms, there is also risk of infections from other patients’ or environmental microbes if hand hygiene measures and other precautions are not ensured. In Pakistan majority of the hospitals do not have any shoe change policy in the intensive care units. Only a small number of hospitals have implemented the use of personal protective measures for infection prevention and restricted access to the intensive care areas.

Earlier studies have shown that the use of barrier nursing and protective measures by the staff in ICU will reduce the incidence of infections due to reduced contamination.^13^ In a related study carried out to find the efficacy of protective footwear on bacterial infection, no significant difference was found in the infection rates with and without the use of protective footwear.^11^^,^^12^ The study done in our setup reached the same inference i.e. not only the use of foot wear has no benefit regarding the control of infections in the critical care but also the rates of infection were more, this was probably due to the fact that individuals while putting on the shoe covers the shoes contaminated their hands and thus further transmitted the infection.

There are several factors other than protective foot wear, in the medical facilities of the developed countries to limit infection rates in ICU. These include measures like strict hand washing and limited numbers of visitors entering the ICU which already reduces the bacterial floor contamination so uncertain measures like use of shoe covers are not considered important in infection control.^14^ Whereas, the hospitals in the developing countries like Pakistan (where the general environment is not clean enough) hand washing is not strictly followed and the number of visitors in ICU cannot be strictly controlled, so use of shoe covers has been used in some hospitals with the idea to control the ICU infection rates and decrease the air and floor colony count but as per the results of this study, no reduction is seen in infection rates, mortality and length of stay by using the shoe covers in intensive care settings.

In a study conducted by Gupta A et al, to find the efficacy of protective footwear on bacterial floor colonization, the floor and air colony counts showed no significant difference in the two phases, with and without protective footwear.^12^ This further rationalizes the adoption of a more restricted access to ICUs in terms of the number of personnel allowed to enter the ICU and at the same time questions the practice of shoe change policy still used in our hospitals.

However, our study had limitations, First it was single centered study and the other hospitals of the territory were not included, Second, sample size of the two groups were varied 55.4% vs 44.6% patients in the shoe cover group, third the patients admitted in ICU have multiple comorbidities in addition to infection which contributed to the mortality, these factors were not considered.

## CONCLUSION

Use of shoe covers in critical care area is not helpful in preventing infections of common ICU pathogens; nor has it decreased the mortality and length of stay in ICU patients. It requires more studies to be carried out involving aspects such as shoe change practices, restricted access, etc.; so that definite policies can be laid down for infection control in critical care patients. While developing such policies, an integrated approach should be undertaken by involving both the administrators and clinicians, in order to achieve optimum results on implementation of the same.
